# Supplementary material for: SyNDI: synchronous network data integration framework
Source: BMC Bioinformatics. 2018 Nov 6;19:403. doi: 10.1186/s12859-018-2426-5 (PMC6219086; doi:10.1186/s12859-018-2426-5)
Supplement: Supplementary file 5 — Files of the example presented in Additional file 4. This additional file is a zip package that contains all files used in Additional file 4. Detailed descriptions of each of these files are presented in a README file included in this zip package. (GZ 30411 kb) [file 12859_2018_2426_MOESM5_ESM.gz › SyNDITools/Meme2Fimo/meme_4.9.0_4.tar.gz!/meme_4.9.0/doc/remove-alignment-gaps.xml]

remove-alignment-gaps


# remove-alignment-gaps

**Usage:** `reduce-alignment-gaps <species> <alignment>`

**Description:**

Remove from an alignment all columns that contain a gap in
the specified species.

**Input:**

- Species name
- A DNA or protein sequence alignment in clustalw format.

**Output:**

- The extracted columns are written to `stdout`.

**Options:**

- None

**Warning messages:**

- None

**Bugs:**
None known.
